# Selective Impairments of Resting-State Networks in Minimal Hepatic Encephalopathy

**DOI:** 10.1371/journal.pone.0037400

**Published:** 2012-05-25

**Authors:** Rongfeng Qi, Long Jiang Zhang, Qiang Xu, Jianhui Zhong, Shengyong Wu, Zhiqiang Zhang, Wei Liao, Ling Ni, Zongjun Zhang, Huafu Chen, Yuan Zhong, Qing Jiao, Xingjiang Wu, Xinxin Fan, Yijun Liu, Guangming Lu

**Affiliations:** 1 Department of Medical Imaging, Jinling Hospital, Clinical School of Medical College, Nanjing University, Nanjing, Jiangsu Province, China; 2 Key Laboratory for NeuroInformation of Ministry of Education, School of Life Science and Technology, University of Electronic Science and Technology of China, Sichuan Province, China; 3 Department of Imaging Sciences, University of Rochester School of Medicine and Dentistry, Rochester, New York, United States of America; 4 Medical Imaging Institute of Tianjin, Tianjin, China; 5 Department of General Surgery, Jinling Hospital, Clinical School of Medical College, Nanjing University, Nanjing, Jiangsu Province, China; 6 Department of Psychiatry, University of Florida McKnight Brain Institute, Gainesville, Florida, United States of America; Banner Alzheimer's Institute, United States of America

## Abstract

**Background:**

Minimal hepatic encephalopathy (MHE) is a neuro-cognitive dysfunction characterized by impairment in attention, vigilance and integrative functions, while the sensorimotor function was often unaffected. Little is known, so far, about the exact neuro-pathophysiological mechanisms of aberrant cognition function in this disease.

**Methodology/Principal Findings:**

To investigate how the brain function is changed in MHE, we applied a resting-state fMRI approach with independent component analysis (ICA) to assess the differences of resting-state networks (RSNs) between MHE patients and healthy controls. Fourteen MHE patients and 14 age-and sex-matched healthy subjects underwent resting-state fMRI scans. ICA was used to identify six RSNs [dorsal attention network (DAN), default mode network (DMN), visual network (VN), auditory network (AN), sensorimotor network (SMN), self-referential network (SRN)] in each subject. Group maps of each RSN were compared between the MHE and healthy control groups. Pearson correlation analysis was performed between the RSNs functional connectivity (FC) and venous blood ammonia levels, and neuropsychological tests scores for all patients. Compared with the healthy controls, MHE patients showed significantly decreased FC in DAN, both decreased and increased FC in DMN, AN and VN. No significant differences were found in SRN and SMN between two groups. A relationship between FC and blood ammonia levels/neuropsychological tests scores were found in specific regions of RSNs, including middle and medial frontal gyrus, inferior parietal lobule, as well as anterior and posterior cingulate cortex/precuneus.

**Conclusions/Significance:**

MHE patients have selective impairments of RSNs intrinsic functional connectivity, with aberrant functional connectivity in DAN, DMN, VN, AN, and spared SMN and SRN. Our fMRI study might supply a novel way to understand the neuropathophysiological mechanism of cognition function changes in MHE.

## Introduction

Hepatic encephalopathy (HE) is a common and serious neuro-cognitive dysfunction in patients with acute and chronic hepatic dysfunction, which is characterized by a wide spectrum of clinical manifestations, ranging from alterations of psychometric performance to stupor and coma [1], [2]. The term minimal hepatic encephalopathy (MHE) is used to classify a subpopulation of cirrhotic patients with no obvious clinical manifestation but can be identified with neuropsychological examination, such as the number connection test and the digit symbol test [3], [4]. MHE, with the prevalence varied between 30% [5] and 84% [6] in cirrhosis patients, has a detrimental effect on health-related quality of life [7], [8], [9], and has some propensity to the development of clinical HE. In previous behavioral studies, MHE was found to prominently affect the cognition function in the domain of attention, vigilance and integrative functions, while the sensory-motor areas were often unaffected [7], [9]. However, the exact pathophysiological mechanisms of these cognition function changes in MHE patients remain unclear so far.

Functional neuroimaging plays an important role in uncovering functional abnormality of the brain in MHE patients. Position emission tomography (PET) and single-photo emission computerized tomography (SPECT) studies have demonstrated that MHE patients exhibited abnormalities in resting cerebral blood flow and cerebral metabolic rate of glucose and ammonia in many brain regions, especially in frontal and parietal cortices [10],[11]. Two prior task-state blood oxygenation level dependent (BOLD) functional MRI (fMRI) studies showed that the MHE patients exhibit abnormal brain activation when performing the task. In the first report, Zafiris *et al.* [12] first analyzed the neural mechanisms of nine non-manifest HE patients using the critical flicker frequency (CFF) test, and found an early-impaired and compensatory neural mechanism during visual judgment in these patients. Additionally, Zhang *et al.* observed an abnormal cognitive control function in a group of cirrhotic patients (half with MHE), using a modified Chinese Stroop task [13].

Recently, fMRI studies have indicated that the pathophysiology of many human brain diseases such as HE may be associated with the changes of spontaneous low-frequency (<0.08 Hz) BOLD fluctuations measured during a resting state. Since Biswal *et al.* [14] showed that spontaneous low-frequency fluctuation is highly synchronous among bilateral motor cortices, abnormal synchronization has been reported in many mental diseases such as Alzheimer's disease [15], [16] and attention deficit hyperactivity disorder (ADHD) [17]. A recent work using independent component analysis revealed reduced resting state synchronization between default mode network in overt hepatic encephalopathy patients [18]. In fact, the effects of HE may be more global [2], however, whether other brain networks are also affected by hepatic encephalopathy has not been fully elucidated previously. Based on the findings in other neurological diseases that abnormal resting-state brain networks (RSNs) already exist in the early stage of disease, e.g., in early Alzheimer's disease [19], we hypothesized that RSNs may also be modified in the mildest form of hepatic encephalopathy-MHE. In addition, in many previous PET or fMRI studies, abnormalities of brain regions devoted to attention, social cognition, and vision functions were found in MHE patients as compared to healthy controls, while the sensory-motor areas were often unaffected [12], [13], [20]. So our second hypothesis is that aberrant RSNs of MHE may mainly occur in networks associated with attentional, social cognitional and visual processing, while the somato-motor network is little affected.

To test our hypotheses, we adopted independent component analysis (ICA) to investigate the changes of functional connectivity (FC) of RSNs in MHE patients. ICA is a model-free analysis technique that can separate a set of spatially independent components from mixed BOLD signals, the brain areas of the same component map were considered as an approximate synchronized network [21].

## Materials and Methods

### Subjects

This study was approved by the Medical Research Ethics Committee of Jinling Hospital and Clinical School of Medical College at Nanjing University. Written informed consents were obtained from all the participants before the study. The MHE patients were recruited from patients hospitalized at Jinling Hospital, Nanjing, China. Fourteen hepatic cirrhosis patients with MHE (11 male, 3 women, mean age: 56.57±9.19 years) were included in this study. The diagnosis of MHE was made according to the recommendation by the working party of 11^th^ world congress of gastroenterology [3]. In our study, all patients underwent two neuropsychological tests: number connecting-A (NCT-A) and digit symbol test (DST). In the NCT-A test, the subjects have to connect 25 numbers printed on paper consecutively from 1 to 25 as quickly as possible, with the test score being the time the patients perform the test. In the DST, the subjects are given a list of digits from 1 to 9 associated with symbols and are required to fill in blanks with symbols that correspond to each number, with the test score being the total number of correct sequential matching of symbols to numbers in a 90-second interval [7]. When the scores of at least one test were beyond 2SD (standard deviation) of mean value of age-matched healthy controls, the cirrhotic patients could be regarding having MHE [7], [22]. The additional inclusion criteria were as follows: (1) no previous history of episodes of HE; (2) no other neuropsychological disorders, trauma, or drug abuse history; (3) head motion less than 1.0 mm; (4) no brain lesions or cerebral vascular disease on conventional MR images. Laboratory parameters including prothrombin time, protein metabolism tests, venous blood ammonia were obtained from all patients to assess the severity of liver disease, within one week before MR scanning. Of these 14 MHE patients, 12 patients had hepatitis B; the other two were alcoholic cirrhotic patients. Twelve patients had Child-Pugh grade A and 2 patients had Child-Pugh grade B. All patients were right-handed. Fourteen age-and gender-matched right-handed healthy controls from local community were recruited in this study (11 male, 3 women, and mean age: 55.00±9.52 years). All healthy controls had no diseases of the liver and other systems, with no abnormal findings in abdominal ultrasound scans and conventional brain MR imaging. All controls underwent neuropsychological tests before the MR scanning. No laboratory tests were performed thus unavailable for them. Demographics and clinical data for all the 28 participants were summarized in Table I.

**Table I:** Demographics and clinical data of HE patients and healthy controls.

Protocols	HC (n = 14)	MHE (n = 14)	*P* value
Sex (M/F)	11/3	11/3	1^a^
Age (±SD), y	55.00±9.52	56.57±9.19	0.66^b^
Education, y	13.36±2.10	13.07±1.59	0.69^b^
Venous blood ammonia (in mol/L)		38.58±25.55	
Child-Pugh scale			
A		12	
B		2	
C			
NCT	45.8±11.7	76.5±14.9	= 0.002^b^
DST	42.0±12.7	27.5±6.2	= 0.002^b^

Values are expressed as mean ± SD. HC = healthy control; MHE = minimal hepatic encephalopathy.

a The *P* value for gender distribution in the two groups was obtained by chi-square test.

b The *P* value for age and neuropsychological tests difference between the two patients groups was obtained by two sample *t* test.

### MRI data acquisition

MRI data were acquired on a 1.5-Tesla Signa scanner (GE Healthcare, Milwaukee, WI, USA) using a standard whole-head coil. The participants were instructed to lie quietly, keep their eyes closed but be awake in the MR scanner. Axial anatomical images were first acquired using a T1-FLAIR sequence: TR/TE = 2000 ms/24 ms, TI = 750 ms, slices = 23, matrix = 512×512, field of view (FOV) = 24×24 cm^2^, slice thickness/gap = 4.0 mm/0.5 mm. Functional images were subsequently obtained at the same orientation and thickness as the anatomical slices with a gradient-recalled echo echo-planar imaging (GRE-EPI) sequence (TR/TE = 2000 ms/40 ms, FA = 80°, matrix = 64×64, FOV = 24×24 cm^2^, slices = 23). The functional scanning measured 210 brain volumes which lasted a total 420 seconds. Finally, images with coronal T2-FLAIR sequence (TR/TE = 3600/90 ms, slices = 20, matrix = 256×256, flip angle = 90°, FOV = 24×24 cm^2^, thickness/gap = 5.0 mm/1.5 mm) were collected.

### Data preprocessing

Data were pre-processed using SPM8 software package (http://www.fil.ion.ucl.ac.uk/spm). The first 10 volumes were excluded for magnetization to reach steady state and allowing adaptation of the subjects to the scanning noise. The remaining 200 consecutive volumes were used for data analysis. Slice-timing adjustment and realignment for head-motion correction were performed, and none of subjects was found to have excessive movement (translation exceeded 1.0 mm or rotation exceeded 1.0°). We also evaluated the group differences in translation and rotation of head motion according to the following formula [23]:
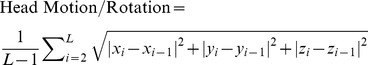
where *L* is the length of the time series (*L* = 200 in this study), *xi*, *yi* and *zi* are translations/rotations at the *i*th time point in the *x*, *y* and *z* directions, respectively. The results showed that the two groups had no significant differences (two sample *t* test, *t* = 1.306, *P* = 0.203 for translational motion and *t* = 1.614, *P* = 0.119 for rotational motion). The functional images were then spatially normalized to stereotaxic coordinates of the standard Montreal Neurological Institute (MNI) and resampled the voxel size into 3×3×3 mm^3^; and then smoothed by convolution with an isotropic Gaussian kernel (FWHW = 8 mm).

### ICA and identification of RSNs

Group spatial independent component analysis (ICA) was performed using the GIFT software (http://icatb.sourceforge.net/, Vision 2.0d). Briefly, ICA separates linear mixed data into spatially independent sources. To determine the number of independent components, dimension estimation on the smoothed data of the two groups was conducted using the minimum description length (MDL) criterion. Then, fMRI data from all subjects in each group were concatenated and the temporal dimension of the aggregate data set was reduced by means of principal component analysis (PCA), respectively, followed by an IC (with time-courses and spatial maps) estimation using the infomax algorithm. Two separate group spatial ICA were separately performed on the MHE and healthy control groups, with 24 and 25 ICs respectively, ensuring that the RSNs had similar spatial pattern in each group. IC time-courses and spatial maps for each subject were back-reconstructed, using the aggregated components and the results from the data reduction step. To each IC, the time courses correspond to the waveform of a specific pattern of coherent brain activity and the intensity of this pattern of brain activity across the voxels was expressed by the associated spatial map [24]. Then, using the GIFT software, the components to be retained for further analysis among the 24/25 estimated ICs were selected based on the largest spatial correlation with specific RSN templates. To display the voxels that contributed most strongly to a particular IC, the intensity values in each spatial map were converted to *z* values. The *z-*values here reflect the degree to which the time courses of a given voxel correlate to the time courses of each special IC. These templates come from our previous studies [25], [26]. Six templates were used in this study: default mode network (DMN), dorsal attention network (DAN), self-referential network (SRN), sensorimotor network (SMN), visual network (VN), and auditory network (AN). DMN [27] is thought to engage in the maintenance of the baseline brain activities related to cognitions of self-awareness, episodic memory and interactive modulation between the internal mind activities and external tasks [21]. DAN [28], a left-lateralized network, is thought to mediate goal-directed top-down processing. SRN [21] may relate to self-referential mental activity. SMN, VN, and AN refer to corresponding sensorimotor, vision, and auditory function respectively [23].

### Group statistical maps

The ICs corresponding to six RSNs were extracted from all subjects, then a second-level random-effects statistical analysis was performed for the each RSN in each group using one-sample *t* test. Significant thresholds were set at a corrected *P*<0.05 [multiple correction using false discovery rate (FDR) criterion]. The group-level RSN maps were then visualized with the BrainNet Viewer (http://www.nitrc.org/projects/bnv/). To compare the RSNs between the MHE and healthy control group, a random-effects analysis two-sample *t* tests were calculated. To take into account of multiple comparisons, the *t*-map of the two-sample *t* test was corrected using AlphaSim program [29] in the REST software [30]. We used a masking procedure to generate six group maps of all 28 subjects corresponding to each RSN (significant thresholds defined as the one-sample *t* test above) and constrained the group comparison result to the voxels within these combined group maps.

### Pearson correlation analyses

To investigate the potential effect of venous blood ammonia on RSNs, and the relationship between RSNs and the neuropsychological tests of MHE patients, the regions within the each RSN that differed significantly between the patients and control groups were extracted as a mask consisting of several regions of interest (ROIs) [23], then the mean *z-*values of each patient within these ROIs were correlated against the venous blood ammonia levels, and the scores of NCT-A and DST, using the Pearson correlation analysis. Correlation analysis was performed using SPSS 16.0 (SPSS Inc., Chicago, IL), the threshold was set at a significance level of *P*<0.05, corrected for multiple comparison using the Bonferroni correction for the number of brain regions that showed aberrant FC.

## Results

### Demographics and clinical data

There were no significant differences in gender, age between MHE and control groups (Table I). However, there were significant differences of neuropsychological scores between patients and controls (NCT-A: *P = *0.002, *t = *3.55; DST: *P = *0.002, *t = *3.43). Compared with the healthy controls, patients with MHE spent more time to complete the NCT-A and had less correct number of DST.

### Spatial pattern of RSNs in each group

**Figure 1:**
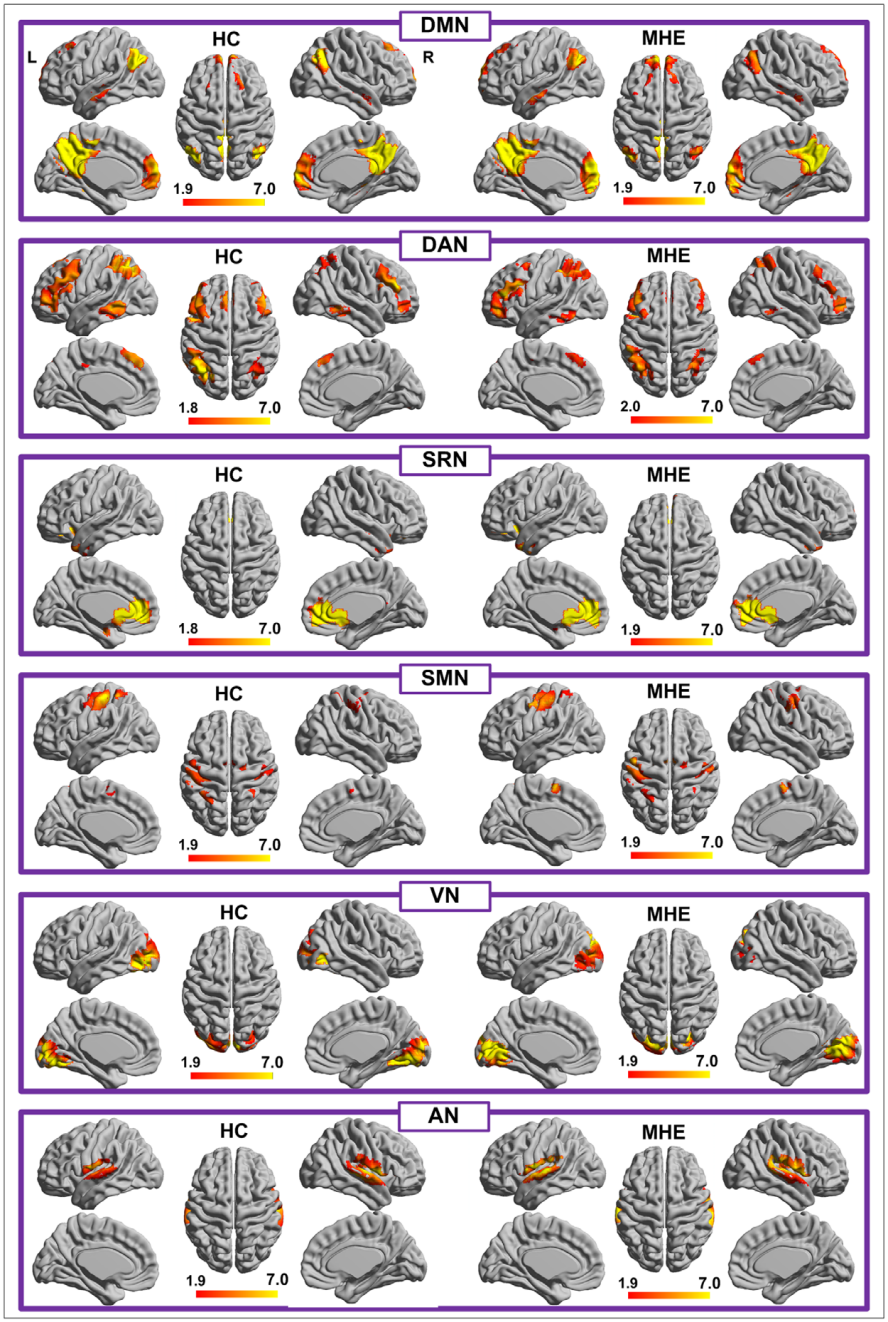
Within group maps of six RSNs in HC and in MHE groups. Lateral and medial views of left hemisphere and lateral and medial views of right hemisphere are shown for each RSN, in addition to the top-down view. The color scale represents T values in each RSN (*P*<0.05, FDR corrected). RSN = resting-state network; HC = healthy control; MHE = minimal hepatic encephalopathy; FDR = false discovery rate; DAN = dorsal attention network; DMN = default mode network; VN = visual network; and AN = auditory network; SMN = sensorimotor network; SRN = self-referential network.

The results of one-sample *t*-tests revealed typical spatial patterns in each RSN of both MHE and control groups, as illustrated in Fig. 1. Among these RSNs, DMN included bilateral angular gyrus, posterior cingulated/precuneus, bilateral superior frontal gyrus, bilateral parahippocampa gyrus, and medial frontal gyrus. DAN primarily included bilateral the intraparietal sulcus, cortex at the intersection of precentral and superior frontal sulcus near the human frontal eye field, ventral precentral, and superior, middle and inferior frontal gyrus. SRN comprised the ventromedial prefrontal cortex (vMPFC), medial orbital prefrontal cortex (MOPFC), gyrus rectus, and pregenual anterior cingulate gyrus (PACC). SMN included precentral, postcentral, the primary sensory-motor cortices, and the supplementary motor area. VN involved the inferior, middle and superior occipital gyrus, the temporal-occipital regions along with superior parietal gyrus. AN primarily included the bilateral middle and superior temporal gyrus (Fig. 1). Thresholds were set at *P*<0.05, corrected with multiple comparisons using the false discovery rate (FDR) criterion.

### Aberrant RSNs in patients with MHE

**Figure 2:**
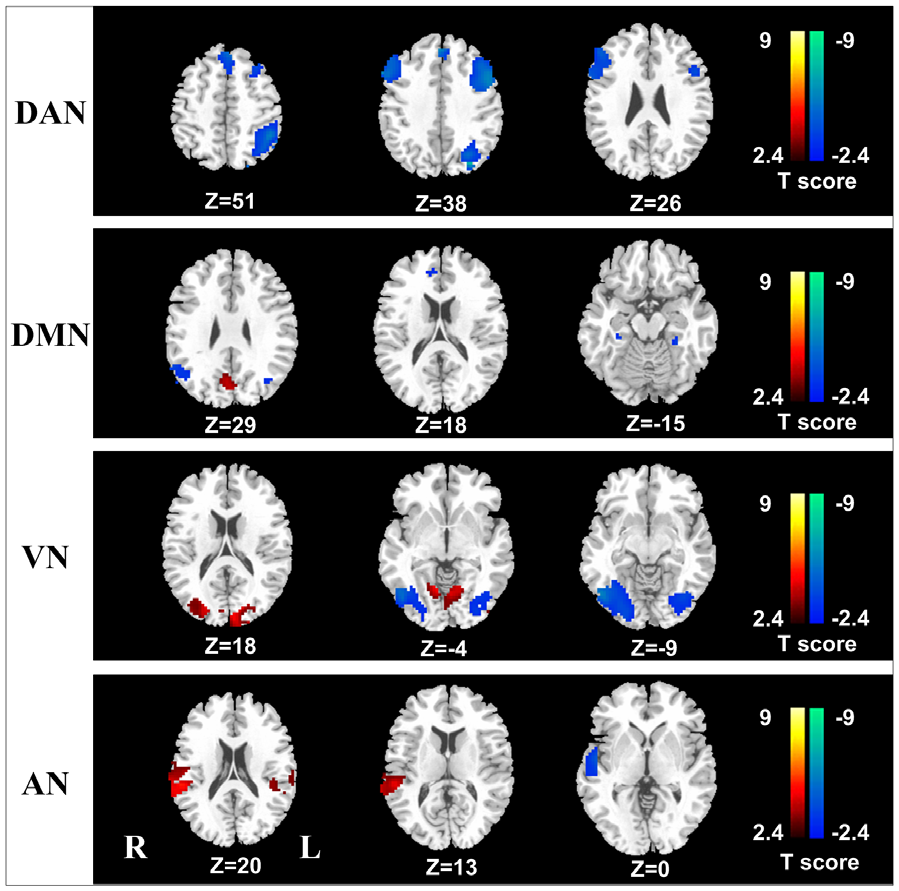
Group comparison maps of six RSNs between MHE patients, and healthy controls. Compared with the healthy controls, MHE patients showed a generally decreased FC within DAN, both decreased and increased FC in DMN, VN, and AN, as well as unchanged SMN and SRN. Significant thresholds were set at a corrected *P*<0.05 using AlphaSim program. The SMN and SN had no statistical significance between the two groups. MHE = minimal hepatic encephalopathy; FC = functional connectivity; DAN = dorsal attention network; DMN = default mode network; VN = visual network; and AN = auditory network; SMN = sensorimotor network; SRN = self-referential network.

The results obtained from the two-sample *t* test clearly showed significant difference of FC between the MHE and healthy controls, setting at a corrected threshold of *P*<0.05 (corrected with Alphasim program) (Fig. 2). Compared with the healthy controls, MHE patients showed a generally decreased FC within DAN, both decreased and increased FC in DMN, VN, and AN, as well as unchanged SMN and SRN. Within these four aberrant RSNs, DAN demonstrated decreased FC in bilateral middle frontal gyrus, bilateral inferior frontal gyrus, medial frontal gyrus, left inferior parietal lobule, bilateral inferior temporal gyrus. DMN displayed decreased FC in bilateral angular gyrus, right anterior cingulate cortex, bilateral parahippocampa gyrus, and increased FC in right posterior cingulate cortex/precuneus. FC of VN decreased in bilateral inferior occipital gyrus and increased in bilateral calcarine fissure. AN showed bilateral (either decreased and increased) changes of FC in right superior temporal gyrus and increased FC in left superior temporal gyrus. See table II for a detail list of brain regions with aberrant FC of each RSN.

**Table II:** Differences of RSNs between MHE patients and healthy controls.

RSNs	Brain regions	Hem	BA	MNI coordinates (mm)	Voxel number	Peak t-score
				(x, y, z)		
DAN	Middle frontal gyrus	L	9, 6	−42, 13, 35	294	−5.29
	Middle frontal gyrus	R	9, 46	51, 20, 34	236	−4.49
	Inferior frontal gyrus	L	44,48	−51, 11,25	114	−3.72
	Inferior frontal gyrus	R	44,48	52, 16,25	81	−4.29
	Medial Frontal Gyrus		8	−90, 38, 43	82	−3.49
	Inferior parietal lobule	L	40, 7	−43, −52, 48	273	−6.71
	Inferior temporal gyrus	L	37, 21	−61, −55, −14	68	−5.58
	Inferior temporal gyrus	R	37, 21	63, −31, −11	41	−4.25
DMN	Angular gyrus	L	39	−33,−76,40	34	−4.72
	Angular gyrus	R	39, 40	51,−70,43	156	−5.01
	Anterior cingulate cortex	R	32, 9	6, 41, 19	25	−2.57*
	Parahippocampa gyrus	L	37, 36	−30, −40, −13	20	−5.27*
	Parahippocampa gyrus	R	37, 36	27, −34, −13	10	−3.72*
	PCC/PCUN		31, 7	0, −43, 40	198	+4.65
VN	Calcarine fissure	L	18,19	−9,−78,7	179	+6.15
	Calcarine fissure	R	18,19	9,−75,7	176	+6.03
	Inferior occipital gyrus	L	19,18	−36,−80,−10	127	−6.34
	Inferior occipital gyrus	R	19,18	36,−76,7	140	−6.27
AN	Superior temporal gyrus	L	40,13	−66, −34, 22	70	+3.46
	Superior temporal gyrus	R	40,22	55,−38,15	133	+5.62
	Superior temporal gyrus	R	22	54,−13,4	143	−2.97

Positive sign in the peak t-score represents increase, and negative sign represents decrease. All *P*<0.05, corrected for multiple comparisons using AlphaSim program. RSN = resting-state network; Hem = hemisphere; BA = Brodmann's area; MHE = minimal HE; MNI = Montreal Neurological Institute. PCC/PCUN = Posterior cingulate cortex/precuneus; DAN = dorsal attention network; DMN = default mode network; VN = visual network; and AN = auditory network.

* The region in the anterior cingulate cortex and bilateral hippocampus survived the height but not the extent threshold.

### Correlation of regions of RSNs with venous blood ammonia and neuropsychological tests scores

**Figure 3:**
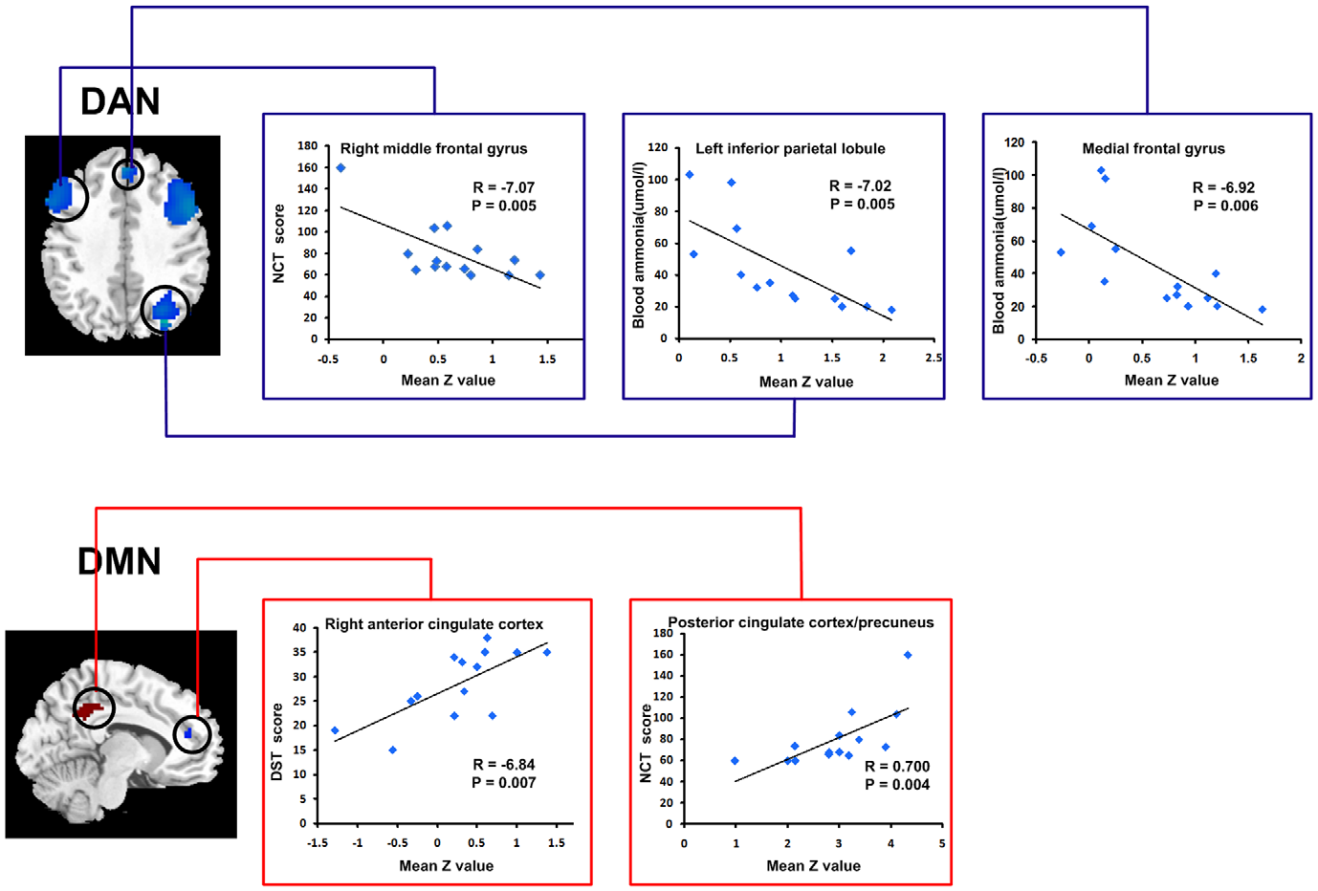
The correlation between venous blood ammonia/neuropsychological tests scores with mean *z*-score of regions that differed significantly between the patient and control groups within each RSN. The venous blood ammonia levels of MHE patients negatively correlated with mean *z-*values of left parietal cortex and medial frontal gyrus within DAN. The DST scores showed positive correlation with mean *z-*values of right anterior cingulate cortex within DMN. The NCT scores demonstrated positive correlation with mean *z-*values of posterior cingulate cortex/precuneus within DMN, and negative correlation with mean *z-*values of right middle frontal gyrus in DAN. (*P*<0.05, Bonferroni corrected). RSN = resting-state network; MHE = minimal hepatic encephalopathy; DAN = dorsal attention network; DMN = default mode network; DST = digit symbol test; NCT = number connection test.

Pearson correlation analyses revealed that the venous blood ammonia levels of MHE patients negatively correlated with mean *z-*values of left parietal cortex and medial frontal gyrus within DAN. The DST scores showed positive correlation with mean *z-*values of right anterior cingulate cortex (ACC) within DMN. The NCT-A demonstrated positive correlation with mean *z-*values of posterior cingulate cortex/precuneus within DMN, and negative correlation with mean *z-*values of right middle frontal gyrus in DAN (corrected *P*<0.05) (Fig. 3). Bonferroni correction was used for the multiple comparisons when performing correlation analysis (8, 6, 4 and 3 brain regions showed aberrant FC in DAN, DMN, VN and AN, respectively. See table II for detail numbers and sizes of brain regions). The other brain regions with aberrant FC showed no significant correlation with neuropsychological tests scores and blood ammonia.

## Discussion

The present fMRI study demonstrated that MHE patients had selectively impaired RSNs. Within six RSNs, the DAN showed decreased FC; the DMN, AN, and the VN displayed a bidirectional change of FC; while the SMN and the SRN were unchanged. We also found a relationship between venous blood ammonia levels/neuropsychological tests scores and FC in some specific regions of RSNs, including the middle and medial frontal gyrus, inferior parietal lobule, as well as anterior and posterior cingulate cortex/precuneus. This resting state fMRI study may be potentially helpful to the understanding of neuro-pathophysiological mechanism of cognition function changes in MHE.

The pattern of dorsal attention network (DAN) obtained in the present study was largely consistent with those mapped with the specific task-induced activation [28]. DAN is involved in voluntary (top-down) orienting and shows activity increases after presentation of cues indicating where, when, or to what subjects should direct their attention. It is also involved in many higher-order cognitive tasks. Attention impairment in MHE patients has been proposed by many epidemiological studies [8], [9], [31]. These attention deficits often in turn lead to learning impairment and difficulties of working memory. The diffusely decreased DAN FC of MHE in the present study is consistent with the disease hypothesis that a defect in attention is a fundamental aspect of this disease. The impaired DAN has not previously been well established in the literature, so our data may contribute to the understanding the attention deficit of MHE.

The DMN abnormalities in HE patients have been reported in some published fMRI studies. In a resting-state fMRI study, Zhang *et al*. first demonstrated a decreased FC of middle frontal gyrus, posterior cingulate cortex/precuneus and angular gyrus within DMN in overt HE patients [18]. A very recent study showed that the impairment of DMN FC still persisted after clinical recovery from previous episodes of overt HE in a group of cirrhotic patients [32]. The findings of present study in decreased FC of right ACC and bilateral angular gurys in the mildest form of HE-MHE are in accordance with previous studies. However, our finding of increased FC of PCC in MHE was inconsistent with previous finding in overt HE. Based on the fact that all MHE patients in this study had no obvious magnification of HE, or any history of episodes of overt HE, we speculate that in the early phase of HE, there might be a compensatory or reorganization mechanism of DMN, an interpretation that is partially supported by the positive correlation between FC of PCC/precuneus and NCT-A scores of patients observed in the present study. The similar compensatory phenomenon was also reported in previous studies [12], [33], e.g., in a task-driven fMRI study Zafiris *et al*. found that when performing the critical flicker frequency task, nonmanifest hepatic encephalopathy patients showed enhanced single activity in the right temporal pole [12]. Validity of this compensatory mechanism interpretation needs to be confirmed in further studies.

Many studies have demonstrated that MHE has a profound negative impact on the ability to drive a car and may be a significant factor behind motor vehicle accidents [31]. The ability to drive a car requires coordination of attention, visual, auditory and vestibular input, which is potentially impaired in MHE patients [9], [31]. Except the decreased DAN FC, the present study also demonstrated both decreased and increased FC in VN and AN, suggesting disturbed cooperation of their corresponding function. Bilateral changes in FC have been reported in many other diseases, e.g., social anxiety disorder [23], and Alzheimer's disease [19]. A decrease in functional activity could be related to functional impairment, while an increase could be interpreted as compensatory reallocation or recruitment of cognitive resource [26], [34]. Compensatory neural mechanism during the visual judgment and resting-state has been reported in nonmanifest hepatic encephalopathy patients [12], [33]. Whether there are also both impairment and compensatory or reorganization mechanism of the RSNs such as VN and AV in MHE patients needs further study in the future. In addition, we found no significant changes of the SMN and SRN in MHE patients when comparing to healthy controls, findings which support the hypothesis of this study that MHE patients have selective impaired RSNs.

In the correlative analysis, we found a negative correlation between blood ammonia of MHE patients and FC of the parietal cortex and medial frontal gyrus within DAN, suggesting functional impairment within DAN may partly arise from an excess of ammonia in the blood. This may be explained by the role of ammonia in the pathogenesis of HE. The degradation of ammonia, a main toxic substance in the brain, results in elevation of the glutamine level in astrocytes, consequently causes both swelling and dysfunction of these cells [2]. The ammonia-related cellular alterations are considered to play an important key in the pathogenesis of MHE [35]. We also found a positive correlation between the DST scores with FC of right ACC in DMN. ACC is a center mediating response selection and allocating attention resources when confronted with competing information-processing streams, it also severs to regular both cognitive and emotional processing [36]. PET studies with ^18^F-fluorodeoxyglucose also showed significant metabolism reductions in the ACC [37]. DST, one of the neuropsychological tests which were most frequently used in defining MHE, mainly tests the domains of attention and psychomotor speed [7]. So the impairment of ACC in MHE patients and positive correlation between DST are quit plausible.

Our study has some limitations. First, sample size for MHE patients is small, with variations in the age and disease on-set time, as well as potential effects of medication such as diuretics for controlling ascites in some patients, which might affect the statistical analysis and results of this study. Further studies in a larger population with similar treatment are needed to verify these findings. Secondly, the ICA method used in the study has its own limitations. Although the RSNs represent a finite set of spatiotemporal basis function from which task-networks are then dynamically assembled and modulated during different behavioral states, their neurophysiological meaning still remains unclear.

In conclusion, we observed selective impairments of RSN intrinsic FC in MHE patients, whose DAN, DMN, VN and AN had aberrant functional connectivity, while SMN, SRN were unaffected. Our fMRI study might potentially supply a novel way to understand the neuro-pathophysiological mechanism of cognition function changes in MHE.
